# A retrospective study of sentinel lymph node biopsy for skin cancer in Japan: Comparison with breast cancer and evaluation of factors related to its use

**DOI:** 10.1002/cam4.6677

**Published:** 2023-10-30

**Authors:** Shogo Wada, Tomone Watanabe, Taisuke Ishii, Yuichi Ichinose, Ryoko Rikitake, Dai Ogata, Eiji Nakano, Kenjiro Namikawa, Naoya Yamazaki, Takahiro Higashi

**Affiliations:** ^1^ Department of Dermatologic Oncology National Cancer Center Hospital Tokyo Japan; ^2^ Division of Health Services Research, Center for Cancer Control and Information Services National Cancer Center Tokyo Japan; ^3^ Department of Public Health and Health Policy, Graduate School of Medicine The University of Tokyo Tokyo Japan

**Keywords:** breast cancer, cancer management, clinical management, melanoma, surgical oncology

## Abstract

**Background:**

Sentinel lymph node biopsy (SLNB) underuse has been reported for skin cancers; however, actual performance rates have not been compared. The objective of this study was to investigate the SLNB performance rate in skin cancers covered by health insurance in Japan and compare it with that in breast cancer.

**Methods:**

This was a retrospective study of the SLNB performance rate in SLNB‐eligible patients with breast or skin cancer from 2018 to 2019, utilizing a database linked to the Hospital‐Based Cancer Registry and Diagnosis Procedure Combination survey. Demographic and tumor characteristics were analyzed using logistic regression.

**Results:**

A total of 71,652 patients were included in this study. SLNB was performed in 86.4% (57,904/67,036) of the patients with breast cancer, 44.7% (694/1552) with melanomas, 3.1% (89/2849) with squamous cell carcinomas (SCCs), and 13.5% (29/215) with Merkel cell carcinomas (MCCs). The performance rate of SLNB was significantly lower for skin cancers than for breast cancers (odds ratio [OR], 0.03; *p* < 0.001). In addition, the performance rates of SLNB were significantly lower for SCCs and MCCs than for melanomas (SCC: OR, 0.04; *p* < 0.001; MCC: OR, 0.19; *p* < 0.001). Factors associated with SLNB performance included age, sex, year of incidence, primary tumor site, T stage, and number of hospital beds.

**Conclusions:**

SLNB is underutilized for skin cancer. Further investigation is required to explore the reasons for its underutilization so that it may be implemented more universally.

## 1 INTRODUCTION

Sentinel lymph node biopsy (SLNB) was developed in the early 1990s as a minimally invasive method to detect occult metastases.^1^ Since then, it has become a widely accepted diagnostic procedure. Multiple studies have shown the significance of SLNB in breast and skin cancers, as it has been helpful in improving regional disease control, reducing unnecessary lymph node dissection, and providing prognostic information by investigating tumor deposits in the sentinel lymph node (SLN) pathologically.^2^, ^3^, ^4^, ^5^ SLNB has been reported to extend the survival of patients with breast cancer and melanoma by detecting occult metastases, while the effect in extending the survival of patients with other skin cancers is not established.^4^, ^6^ In addition, since the development of effective systemic therapy, SLNB has recently gained attention for identifying patients who may benefit from adjuvant systemic therapy.^7^

In Japan, the national health insurance has covered SLNB for breast cancer and melanoma since 2010, SLNB for squamous cell carcinoma (SCC) and Merkel cell carcinoma (MCC) since 2018, and SLNB for extramammary Paget's disease since 2020.

Despite the usefulness of SLNB, its underuse has been reported in previous studies. Previous studies have reported SLNB usage rates in breast cancer, melanoma, and MCC as 77%–92%, 48%–76%, and 52.7%, respectively.^8^ The underutilization of SLNB can cause preventable distant metastases and unnecessary lymph node dissection. Thus, it is necessary to investigate the reason for the underutilization and research the precise figure of the SLNB performance rate. However, previous studies have focused only on single types of cancer, and the periods of study and populations of patients were not uniform. Therefore, previous data on SLNB performance cannot be compared to know accurately for which type of cancer SLNB is underused. Additionally, the performance of SLNB in SCC has not yet been reported. The lacking data prevent us from knowing the reality of SLNB underutilization. This study aimed to evaluate the rates of SLNB usage in patients with breast cancer, melanomas, SCCs, and MCCs and analyze the factors associated with its performance using a national database. We emphasize the importance of recognizing the real practice of SLNB and the reasons for the underutilization.

## 2 MATERIALS AND METHODS

### 2.1 Data source

We used a Japanese database that linked the national database of the Hospital‐Based Cancer Registry (HBCR)^9^ with health insurance claims‐equivalent data from the Diagnosis Procedure Combination (DPC) survey. The HBCR system is mandated in all designated cancer care hospitals, and voluntarily operates in non‐designated hospitals with similar roles in their communities. The HBCR data include clinical details such as the Union for International Cancer Control clinical and pathological stages, tumor‐node‐metastasis (TNM) classifications, tumor location, and histopathological findings based on the International Classification of Diseases Oncology third edition (ICD‐O‐3.1). The DPC survey data contain information on all health services provided. Although the DPC system is a grouping system used to determine the overall amount of per‐day health insurance reimbursement to hospitals, the DPC survey data contain equivalent information for fee‐for‐service claims, including individual tests, images, procedures, and prescription drugs, along with service dates and unit costs from both inpatient and outpatient settings. A total of 613 hospitals in Japan were included in this study. All hospitals had more than 100 beds, with size ranging from community hospitals to high‐volume centers. We collected the utilization data from October 2017 to March 2021 and linked them to the HBCR data. The period for DPC data collection was selected to allow the inclusion of all treatments performed for cancers diagnosed between January 2018 and December 2019, when the National Comprehensive Cancer Network (NCCN) guidelines for SLNB remained consistent throughout the study period.

### 2.2 Patient selection

Patients with breast cancer, melanomas, SCCs, and MCCs were selected according to the ICD‐O‐3.1. Patients with breast cancers were selected using topology code C50 and morphology codes 8000–8158, 8160–8162, 8170–8231, 8246/32–8247/39, 8250–8551, 8560–8576, 8940–8941, 8982, and 8980–8981. Patients with melanomas were selected using the topology codes C44, C51, C60.9, and C63.2, and morphology codes 8720–8723, 8730, 8740–8746, 8761, and 8770–8774. Patients with SCCs were selected using the topology codes C44, C51, C60.9, and C63.2, and the morphology codes 8050–8053, 8070–8078, 8083, and 8084. Patients with MCCs were selected using the topology codes C44, C51, C60.9, and C63.2, and the morphology code 8247.

We included patients eligible for SLNB coverage under the Japanese health insurance. We excluded patients suspected to have nodal or distant metastases at diagnosis; patients with tumors in situ of breast cancer, melanoma, and SCC; and patients with T4 breast cancer and T1 SCC (<2 cm). Patients with incomplete data were also excluded from the analysis.

### 2.3 Variables

Demographic, tumor, and treatment characteristics were derived from HBCR data. The demographic information included sex and age at the time of diagnosis. Tumor characteristics included the location of the primary tumor, TNM stage, and histology. Treatment characteristics included the type of surgery, date of the procedure, and number of beds in the hospital where the procedure was performed.

### 2.4 Statistical analysis

SLNB usage performance was defined as the ratio of the number of patients who underwent SLNB to the number of SLNB‐eligible patients. Univariate analysis was performed to determine factors individually associated with SLNB performance. In addition, multivariate analysis was conducted to compare the SLNB performance between breast and skin cancers (melanoma, SCC, and MCC) and between melanoma and other skin cancers (SCC and MCC) by adjusting for confounding factors.

Descriptive statistics were presented as frequencies for categorical variables and medians and interquartile ranges for continuous variables. Patients were grouped by age as <65, 65–75, and >75 years to evaluate the effects of advanced patient age. The tumor sites were classified as head/neck, trunk, upper extremity, and lower extremity. The number of hospital beds was grouped into <500 and ≥500 beds to investigate the association between hospital size and the performance of SLNB for breast or skin cancer. Univariate analyses were performed using Pearson's chi‐squared test for categorical variables and Wilcoxon's rank‐sum test for continuous variables. Demographic information, tumor characteristics, and treatment characteristics were analyzed using univariate and multivariate logistic regression analyses. All the tests were two‐sided. Statistical analyses were performed using StataCorp version 17 (StataCorp LLC). The study protocol was approved by the Institutional Review Board of the National Cancer Center, Japan (approval No. 2013‐081, 2022‐275, 2023‐J‐3).

## 3 RESULTS

The demographic and clinical characteristics of the SLNB‐eligible patients with breast cancer, melanoma, SCCs, and MCCs are summarized in Table 1. In total, 71,652 patients (median [IQR] age, 63 [50–73] years; 68,844 [96.1%] female patients) were included in this study. Of the SLNB‐eligible patients for each cancer type, 86.4% of breast cancer patients, 44.7% of melanoma patients (57.0% of T2–3 melanoma patients), 3.1% of patients with SCCs, and 13.5% of patients with MCCs, underwent SLNB.

**TABLE 1:** Comparison of demographic and clinical characteristics of the cancer patients.

	Breast cancer (*n* = 67,036)			Melanoma (*n* = 1552)			SCC (*n* = 2849)			MCC (*n* = 215)		
	SLNB performed (*n* = 57,904, 86.4%) *N* (%)	SLNB not performed (*n* = 9132, 13.6%) *N* (%)	*p* value	SLNB performed (*n* = 694, 44.7%) *N* (%)	SLNB not performed (*n* = 858, 55.3%) *N* (%)	*p* value	SLNB performed (*n* = 89, 3.1%) *N* (%)	SLNB not performed (*n* = 2760, 96.9%) *N* (%)	*p* Value	SLNB performed (*n* = 29, 13.5%) *N* (%)	SLNB not performed (*n* = 186, 86.5%) *N* (%)	*p* value
Age at diagnosis (median, y, IQR)	62 (50–72)	70 (56–82)	**<0.001**	71 (60–78)	77 (66–84)	**<0.001**	71 (64–79)	85 (76–90)	**<0.001**	77 (72–84)	86 (80–91)	**<0.001**
Age at diagnosis (y, *n* [%])												
<65	32,034 (90.4)	3421 (9.6)	**<0.001**	223 (52.7)	200 (47.3)	**<0.001**	23 (8.4)	250 (91.6)	**<0.001**	1–3	4–6	**0.002**
65–75	16,250 (88.8)	2051 (11.2)		238 (53.8)	204 (46.2)		32 (7.5)	396 (92.5)		11 (28.9)	27 (71.1)	
>75	9620 (72.4)	3660 (27.6)		233 (33.9)	454 (66.1)		34 (1.6)	2114 (98.4)		16 (9.4)	155 (90.6)	
Sex (*n* [%])												
Male	322 (75.4)	105 (24.6)	**<0.001**	351 (48.0)	380 (52.0)	**0.014**	66 (4.2)	1494 (95.8)	**<0.001**	15 (16.7)	75 (83.3)	0.247
Female	57,582 (86.5)	9027 (13.5)		343 (41.8)	478 (58.2)		23 (1.8)	1266 (98.2)		14 (11.2)	111 (88.8)	
Incidence year (*n* [%])												
2018	27,948 (86.6)	4334 (13.4)	0.152	340 (45.6)	406 (54.4)	0.512	45 (3.1)	1405 (96.9)	**0.004**	7–9	96	**0.039**
2019	29,956 (86.2)	4798 (13.8)		354 (43.9)	452 (56.1)		44 (3.2)	1355 (96.8)		20 (18.2)	90 (81.8)	
Tumor site (*n* [%])												
Head and neck				91 (26.8)	249 (73.2)	**<0.001**	27 (1.6)	1640 (98.4)	**<0.001**	16 (10.3)	139 (89.7)	0.108
Trunk	57,904 (86.4)	9132 (13.6)		112 (45.3)	135 (54.7)		16 (4.0)	380 (96.0)		1–3	4–6	
Upper extremity				118 (42.3)	161 (57.7)		13 (4.4)	285 (95.6)		4–6	25	
Lower extremity				373 (54.5)	311 (45.5)		33 (6.8)	453 (93.2)		4–6	16	
T stage (*n* [%])												
T1	38,506 (88.0)	5268 (12.0)	**<0.001**	140 (30.7)	316 (69.3)	**<0.001**				15 (12.9)	101 (87.1)	0.909
T2	18,597 (84.0)	3545 (16.0)		166 (58.7)	117 (41.3)		56 (2.7)	2003 (97.3)	**0.007**	10 (14.7)	58 (85.3)	
T3	795 (71.7)	314 (28.3)		182 (55.5)	146 (44.5)		33 (5.2)	597 (94.8)		1–3	15	
T4				197 (47.0)	222 (53.0)		0 (0)	12 (100.0)		1–3	11	
Hospital beds (median, *n*, IQR)	566 (430–713)	520 (391–648)	**<0.001**	760 (584–890)	626 (518–815)	**<0.001**	792 (578–930)	588 (470.5–760)	**<0.001**	677 (455–927)	597.5 (475–772)	0.194
Hospital beds (*n* [%])												
<500	23,174 (84.5)	4261 (15.5)	**<0.001**	85 (29.6)	202 (70.4)	**<0.001**	7–9	851	**<0.001**	7–9	56	0.09
500–750	22,297 (86.5)	3491 (13.5)		253 (41.0)	364 (59.0)		31 (2.5)	1199 (97.5)		7–9	76	
>750	12,433 (90.0)	1380 (10.0)		356 (54.9)	292 (45.1)		49 (6.5)	710 (93.5)		14 (20.6)	54 (79.4)	

*Note*: Bold values indicate statistical significance (*p* < 0.05).

Abbreviations: IQR, interquartile range; MCC, Merkel cell carcinoma; SCC, squamous cell carcinoma; SLNB, sentinel lymph node biopsy; y, years.

The factors that influenced SLNB performance in SLNB‐eligible patients and a comparison of SLNB performance between breast and skin cancers (melanoma, SCC, and MCC) are summarized in Table 2. On univariate analysis, SLNB was performed less frequently in patients with skin cancers (odds ratio [OR] 0.03, 95% confidence interval [CI]: 0.03–0.04, *p* < 0.001) and more frequently in patients aged ≤75 years (OR 3.98, 95% CI: 3.78–4.19, *p* < 0.001 for those aged 65–75 years; and OR 5.37, 95% CI: 5.13–5.62, *p* < 0.001 for those aged <65 years), female patients (OR 14.51, 95% CI: 13.32–15.81, *p* < 0.001), and patients treated in hospitals with ≥750 beds (OR 1.22, 95% CI: 1.15–1.28, *p* < 0.001). On multivariate analysis, SLNB was performed less frequently in patients with skin cancers (OR 0.05, 95% CI: 0.04–0.05, *p* < 0.001) and more frequently in patients aged ≤75 years (OR 3.08, 95% CI: 2.91–3.27, *p* < 0.001 for those aged 65–75 years; and OR 3.56, 95% CI: 3.39–3.75, *p* < 0.001 for those aged <65 years) and patients treated in hospitals with ≥500 beds (OR 1.16, 95% CI: 1.10–1.21, *p* < 0.001 for hospitals with 500–750 beds; and OR 1.66, 95% CI: 1.56–1.77, *p* < 0.001 for hospitals with >750 beds).

**TABLE 2:** Factors influencing performance of sentinel lymph node biopsy in eligible patients: comparison between breast cancer and skin cancer.

	Frequency of performing SLNB in eligible patients				
		Univariate analysis		Multivariate analysis	
	No/Total No (%)	OR (95% CI)	*p* value	OR (95% CI)	*p* value
Cancer type					
Breast cancer	57,904/67,036 (86.4)	1 [Reference]		1 [Reference]	
Skin cancer (Melanoma, SCC, MCC)	812/4616 (17.6)	0.03 (0.03–0.04)	**<0.001**	0.05 (0.04–0.05)	**<0.001**
Age at diagnosis (y)					
>75	9903/16,286 (60.8)	1 [Reference]		1 [Reference]	
65–75	16,531/19,209 (86.1)	3.98 (3.78–4.19)	**<0.001**	3.08 (2.91–3.27)	**<0.001**
<65	32,282/36,157 (89.3)	5.37 (5.13–5.62)	**<0.001**	3.56 (3.39–3.75)	**<0.001**
Sex					
Male	754/2808 (26.9)	1 [Reference]		1 [Reference]	
Female	57,962/68,844 (84.2)	14.51 (13.32–15.81)	**<0.001**	1.13 (0.99–1.29)	0.076
Incidence years					
2018	28,342/34,583 (82.0)	1 [Reference]		1 [Reference]	
2019	30,374/37,069 (81.9)	1.00 (0.96–1.04)	0.96	0.99 (0.95–1.04)	0.737
Hospital beds					
<500	23,276/28,646 (81.3)	1 [Reference]		1 [Reference]	
500–750	22,588/27,718 (81.5)	1.02 (0.97–1.06)	0.468	1.16 (1.10–1.21)	**<0.001**
>750	12,852/15,288 (84.1)	1.22 (1.15–1.28)	**<0.001**	1.66 (1.56–1.77)	**<0.001**

*Note*: Bold values indicate statistical significance (*p* < 0.05). Hospital beds refer to the number of available beds in the hospital where the SLNB was performed.

Abbreviations: CI, confidence interval; MCC, Merkel cell carcinoma; OR, odds ratio; SCC, squamous cell carcinoma; SLNB, sentinel lymph node biopsy; y, years.

The factors related to SLNB performance in SLNB‐eligible patients and a comparison of SLNB performance among melanoma, SCC, and MCC are summarized in Table 3. On univariate analysis, SLNB was significantly performed less frequently in patients with SCCs and MCCs (OR 0.04, 95% CI: 0.03–0.05, *p* < 0.001 for SCC; and OR 0.19, 95% CI: 0.13–0.29, *p* < 0.001 for MCC) and head/neck tumors (OR 0.26, 95% CI: 0.20–0.34, *p* < 0.001). By contrast, SLNB was performed more frequently in patients aged ≤75 years (OR 4.31, 95% CI: 3.58–5.20, *p <* 0.001 for those aged 65–75 years; and OR 5.26, 95% CI: 3.31–6.40, *p* < 0.001 for those aged <65 years), patients with tumors located in lower extremities (OR 2.12, 95% CI: 1.69–2.65, *p* < 0.001), and patients treated in hospitals with ≥500 beds (OR 1.93, 95% CI: 1.52–2.45, *p* < 0.001 for hospitals with 500–750 beds; and OR 4.31, 95% CI: 3.42–5.44, *p* < 0.001 for hospitals with >750 beds). On multivariate analysis, SLNB was significantly performed less frequently in patients with SCCs and MCCs (OR 0.06, 95% CI: 0.05–0.08, *p* < 0.001 for SCC; and OR 0.42, 95% CI: 0.27–0.68, *p* < 0.001 for MCC), female patients (OR 0.69, 95% CI: 13.32–15.81, *p* < 0.001), and patients with head/neck tumors (OR 0.51, 95% CI: 0.38–0.70, *p* < 0.001). By contrast, SLNB was performed more in patients aged ≤75 years (OR 2.47, 95% CI: 1.97–3.08, *p* < 0.001 for those aged 65–75 years; and OR 2.19, 95% CI: 1.73–2.78, *p* < 0.001 for those aged <65 years), patients with tumor located in lower extremities (OR 1.67, 95% CI: 1.27–2.20, *p* < 0.001), and patients treated in hospitals with ≥500 beds (OR 1.67, 95% CI: 1.27–2.20, *p* < 0.001 for hospitals with 500–750 beds; and OR 3.11, 95% CI: 2.37–4.08, *p* < 0.001 for hospitals with >750 beds).

**TABLE 3:** Factors influencing performance of sentinel lymph node biopsy in eligible patients: comparison between three types of skin cancer.

	Frequency of performing SLNB in eligible patients				
		Univariate analysis		Multivariate analysis	
	No/Total No (%)	OR (95% CI)	*p* value	OR (95% CI)	*p* value
Cancer type					
Melanoma	694/1552 (44.7)	1 [Reference]		1 [Reference]	
SCC	89/2849 (3.1)	0.04 (0.03–0.05)	**<0.001**	0.06 (0.05–0.08)	**<0.001**
MCC	29/215 (13.5)	0.19 (0.13–0.29)	**<0.001**	0.42 (0.27–0.65)	**<0.001**
Age at diagnosis (y)					
>75	283/3006 (9.4)	1 [Reference]		1 [Reference]	
65–75	281/908 (31.0)	4.31 (3.58–5.20)	**<0.001**	2.47 (1.97–3.08)	**<0.001**
<65	248/702 (35.3)	5.26 (4.31–6.40)	**<0.001**	2.19 (1.73–2.78)	**<0.001**
Sex					
Male	432/2381 (18.1)	1 [Reference]		1 [Reference]	
Female	380/2235 (17.0)	0.92 (0.79–1.08)	0.309	0.69 (0.57–0.84)	**<0.001**
Incidence years					
2018	1197/2381 (50.3)	1 [Reference]		1 [Reference]	
2019	1118/2235 (50.2)	1.07 (0.92–1.24)	**0.044**	0.99 (0.82–1.19)	0.902
Tumor site					
Trunk	130/651 (20.0)	1 [Reference]		1 [Reference]	
Head and neck	134/2162 (6.2)	0.26 (0.20–0.34)	**<0.001**	0.51 (0.38–0.70)	**<0.001**
Upper extremity	136/607 (22.4)	1.16 (0.88–1.52)	0.291	0.97 (0.70–1.34)	0.841
Lower extremity	412/1192 (34.6)	2.12 (1.69–2.65)	**<0.001**	1.67 (1.27–2.20)	**<0.001**
Hospital beds					
<500	102/1211 (8.4)	1 [Reference]		1 [Reference]	
500–750	291/1930 (15.1)	1.93 (1.52–2.45)	**<0.001**	1.67 (1.27–2.20)	**<0.001**
>750	419/1475 (28.4)	4.31 (3.42–5.44)	**<0.001**	3.11 (2.37–4.08)	**<0.001**

*Note*: Bold values indicate statistical significance (*p* < 0.05). Hospital beds refer to the number of available beds in the hospital where the SLNB was performed.

Abbreviations: CI, confidence interval; MCC, Merkel cell carcinoma; OR, odds ratio; SCC, squamous cell carcinoma; SLNB, sentinel lymph node biopsy; y, years.

## 4 DISCUSSION

In this study, we identified the factors influencing SLNB performance and the differences in SLNB performance between breast cancer, melanoma, SCC, and MCC using multivariate analysis. To the best of our knowledge, this is the first study to compare SLNB performance across these four types of cancer. We found that SLNB was much more underutilized among patients with skin cancer than among patients with breast cancer. Factors associated with SLNB performance included age, sex, year of incidence, primary tumor site, T stage, and number of hospital beds. Even after adjusting for factors that influenced SLNB performance, differences in SLNB performance between breast and skin cancers as well as between melanomas and other skin cancers, remained.

Our study showed differences in SLNB performance rates between breast and skin cancers as well as between melanoma and SCC/MCC. These differences are consistent with the varying extents of SLNB recommendations for each cancer type, as outlined in the NCCN guidelines. The NCCN guidelines recommend SLNB for breast cancer if ≤2 positive lymph nodes are confirmed at diagnosis, apart from ductal carcinoma in situ, locally advanced (T4) breast cancer, and inflammatory breast cancer.^10^ As for melanoma, the NCCN guidelines recommend that clinicians should “discuss and consider” SLNB for T1 and T4 melanoma (≤1.0 or >4.0 mm) and “discuss and offer” SLNB for T2 and T3 melanoma (>1.0–4.0 mm), if nodal or distant metastasis is not clinically detected at diagnosis.^11^ On the other hand, SLNB for SCC is basically not recommended in the NCCN guidelines; they recommend that clinicians could discuss and consider SLNB only for very high‐risk SCC (>4 cm, histopathologically poorly differentiated, thickness >6 mm, invasion beyond subcutaneous fat, perineural involvement deeper than the dermis, or lymphatic/vascular involvement).^12^ Regarding MCC, the effectiveness of SLNB is acknowledged, and the NCCN guidelines recommend SLNB for MCC if nodal or distant metastasis is not clinically detected at diagnosis.^13^ There is no clear benchmark for optimal SLNB performance rate, but ideally, SLNB should be performed for breast cancer without distant metastasis (if ≤2 positive lymph nodes are confirmed at diagnosis, apart from ductal carcinoma in situ, locally advanced breast cancer, and inflammatory breast cancer) and T2–3 melanoma without nodal or distant metastasis. Previous studies show SLNB performance rates for breast cancer varying from 19%–91%, so the rate of 86.4% from our study shows that SLNB is appropriately performed in Japan compared to other countries.^14^, ^15^, ^16^, ^17^, ^18^, ^19^, ^20^, ^21^ On the other hand, the rate of 57.0% for T2–3 melanoma is relatively low compared to other countries (23%–88%).^22^, ^23^, ^24^, ^25^, ^26^, ^27^, ^28^, ^29^, ^30^, ^31^, ^32^

We identified some reasons for the underutilization. Low SLNB performance rates in older patients may be attributed to comorbidities that preclude general anesthesia.^8^, ^28^, ^29^ Moreover, the reduced likelihood of SLN positivity with increasing age suggests that older patients are less likely to benefit from SLNB.^33^, ^34^ Our results suggest that sex may also be associated with SLNB performance, which has not been previously reported. Further studies are needed to clarify why female patients with breast cancer and male patients with skin cancer are more likely to undergo SLNB. Regarding the incidence year, SLNB was performed more in patients with SCCs and MCCs in 2019 than in 2018. SLNB for SCC and MCC has been covered by the Japanese insurance since 2018; therefore, more clinicians may have recognized this new coverage in 2019 than in 2018. For tumors in the head/neck, upper extremities, and lower extremities, SLNs are usually located in the neck, axilla, and groin, respectively. For tumors in the trunk, SLNs may be located in any of these areas. SLNB for head/neck tumors may be underutilized because of the complex lymphatic drainage patterns, which make SLNB technically more challenging, less accurate.^35^ Additionally, the SLN is easier to detect in the inguinal area, which may explain the high SLNB performance for tumors in the lower extremities. SLNB is performed more frequently in hospitals with more beds, which could be due to high‐volume hospitals serving as academic centers and having more medical staff to complete SLNB. Previous studies have reported higher compliance with SLNB guidelines in academic facilities.^22^, ^31^, ^36^, ^37^ The increased performance at academic centers is likely due to greater awareness of guidelines in this setting.

There might be other factors contributing to the low SLNB performance rate for skin cancer that could not be detected in our study. Previous studies in the United States showed that low socioeconomic status can prevent patients from accessing medical facilities and receiving proper procedures.^22^, ^31^ However, in Japan, all citizens are covered by the national health insurance in contrast to the United States. In Germany where all citizens are covered by national health insurance, SLNB performance rate for melanoma is 88% and much higher than that in Japan. Therefore, low socioeconomic status has a small effect on SLNB underutilization in Japan.

Considering the difference in SLNB performance rate between breast cancer and skin cancer, two explanations can be listed. Firstly, Japanese dermatologists can be ignorant about the importance of SLNB. According to a previous study, only 56% of Australian dermatologists thought SLNB had an important role in melanoma management.^38^ Another study showed overtreatment or undertreatment of melanoma is more likely to be performed by physicians aged 65+ years and physicians who treated one to five melanoma patients a year.^27^ This tendency can also be seen in Japan. In addition, Japanese guidelines for melanoma management propose no articulated criterion for the selection of patients suitable for SLNB. The difference between the high SLNB performance rate in Germany and the low rate in the Netherlands was attributed to clear guideline recommendations in a previous study.^25^ Therefore, the unclear recommendation for SLNB can also cause the misunderstanding from Japanese dermatologists. Further investigation of the cognition of SLNB by Japanese clinicians is warranted.

Our study was limited by the variables available in the database. We can hypothesize that other reasons for the underutilization of SLNB for skin cancer include comorbidities, patient preference, proficiency, and experience of clinicians, and the availability of medical devices or tracers necessary for SLNB. However, we could not prove these hypotheses because of the lack of associated granular variables. To overcome this limitation, additional studies focusing on collecting more detailed data related to the underutilization of SLNB for skin cancer are necessary. This could involve specific data collection surveys that target variables identified as potential factors for underutilization. Moreover, our study was limited by the possibility of coding errors and missing data. However, this was balanced by the large sample size and comprehensive review of patients.

## 5 CONCLUSIONS

This is the first study to prove SLNB underutilization in skin cancer by directly comparing it with breast cancer. Our study is significant in revealing this critical issue because SLNB underutilization has a negative prognostic impact, especially for melanoma patients. Furthermore, there is variation in SLNB use across histopathological types of skin cancers. Age, sex, year of incidence, primary tumor site, T stage, and hospital size are associated with these differences, suggesting that other factors may also be involved. Our study cautions clinicians about the importance of SLNB and prompts more appropriate usage. Given the recent recognition of the significance of SLNB, further investigation is required to explore the reasons for its underutilization so that it may be implemented more universally to provide standardized care for skin cancers.

## AUTHOR CONTRIBUTIONS

**Shogo Wada:** Conceptualization (lead); data curation (lead); investigation (lead); methodology (supporting); visualization (lead); writing – original draft (lead); writing – review and editing (supporting). **Tomone Watanabe:** Funding acquisition (supporting); methodology (lead); project administration (lead); supervision (lead); writing – review and editing (lead). **Taisuke Ishii:** Formal analysis (supporting); methodology (supporting); project administration (supporting); supervision (supporting); validation (supporting); writing – review and editing (supporting). **Yuichi Ichinose:** Funding acquisition (supporting); project administration (supporting); resources (supporting); supervision (supporting); writing – review and editing (supporting). **Ryoko Rikitake:** Formal analysis (supporting); methodology (supporting); project administration (supporting); supervision (supporting); validation (supporting); writing – review and editing (supporting). **Dai Ogata:** Conceptualization (supporting); supervision (supporting); writing – review and editing (supporting). **Eiji Nakano:** Conceptualization (supporting); supervision (supporting); writing – review and editing (supporting). **Kenjiro Namikawa:** Conceptualization (supporting); supervision (supporting); writing – review and editing (supporting). **Naoya Yamazaki:** Conceptualization (supporting); supervision (supporting); writing – review and editing (supporting). **Takahiro Higashi:** Conceptualization (supporting); data curation (lead); formal analysis (lead); funding acquisition (lead); methodology (lead); project administration (supporting); resources (lead); software (lead); supervision (lead); validation (lead).

## FUNDING INFORMATION

This work was partly supported by the National Cancer Center Research and Development Fund (2013‐081, 2022‐275, 2023‐J‐3).

## CONFLICT OF INTEREST STATEMENT

The authors declare no conflict of interest.

## Data Availability

Data available on request due to privacy/ethical restrictions.
